# Electro osmotically interactive biological study of thermally stratified micropolar nanofluid flow for Copper and Silver nanoparticles in a microchannel

**DOI:** 10.1038/s41598-023-51017-z

**Published:** 2024-01-04

**Authors:** Noreen Sher Akbar, Maimona Rafiq, Taseer Muhammad, Metib Alghamdi

**Affiliations:** 1 DBS&H, CEME, National University of Sciences and Technology, Islamabad, Pakistan; 2 Department of Mathematics, COMSATS University Islamabad, Attock, 43600 Pakistan; 3 Department of Mathematics, College of Science, King Khalid University, Abha, 61413 Saudi Arabia

**Keywords:** Biophysics, Mathematics and computing

## Abstract

A novel mathematical analysis is established that summits the key features of peristaltic propulsion for a non-Newtonian micropolar fluid with the electroosmosis and heat transfer enhancement using nanoparticles. In such physiological models, the channel have a symmetric configuration in accordance with the biological problem. Being mindful of this fact, we have disclosed an integrated analysis on symmetric channel that incorporates major physiological applications. The creeping flow inference is reviewed to model this realistic problem. Flow equations are model using cartesian coordinates and simplified using long wave length and low Reynolds number approximation. Nonlinear linear couple equations are solving numerically. We have studied the variation in the properties of nanofluid developed by two different types of nanoparticles (i.e. Cu and Ag nanoparticles). Graphical illustrations are unveiled to highlight the physical aspects of nanoparticles and flow parameters. The exploration demonstrates that the micro-rotation of the nano-liquid elements enhances the thermal conductivity of the fluid movement. The effect of micropolar fluid parameters on mean flow and pressure variables is also presented.

## Introduction

Peristalsis is a coordinated and rhythmic muscular contraction and relaxation of the walls of certain tubular organs, particularly the digestive and reproductive tracts. This physiological process is essential for the movement of substances, such as food in the digestive system or the passage of gametes in the reproductive system. Peristaltic waves propel the contents through the tubular structures, creating a wave-like motion that helps facilitate the transport of materials from one end to another^1^. It is a vital mechanism in various biological processes, ensuring the proper functioning of organs and systems within the human body. The category of polar fluids with microstructure includes micropolar fluids. The concepts and practical implications of the Navier–Stokes framework for ordinary fluids are significantly generalized by the corresponding model of micropolar fluids. These fluids replicate fluids made up of stiff particles embedded in a viscous medium without taking into account the fluid particles' ability to deform. Since no single universal equation demonstrates all the characteristics of a non-Newtonian fluid model, research on non-Newtonian fluids has led to the study of several fluid models. When C. A. Eringen expanded on his research in the area of microfluidics^2^, he first developed a mathematical representation of micropolar fluids. Numerous research studies have already been conducted on micropolar fluid flow^3–7^.

To transfer food, fluids, and waste, biological systems are dependent on peristaltic travel, which requires the wavelike contractions and relaxations of flexible conduits like the esophagus, intestines, and blood vessels. One of the many areas where peristaltic transport has found significant application over the years is in pharmaceutical industries, microfluidic systems, and pipeline distribution of slurries, to name just a few. The peristaltic motion of different types of fluid has been a deep research area for many researchers during the past few decades. After the introduction of micropolar fluids, the peristaltic motion of this new type of fluid was studied by Devi and Devanathan^8^. Their work was later extended by Srinivasacharya et al.^9^ by incorporating the tube effects on peristaltic pumping of micropolar fluid. In a subsequent study, Hayat et al.^10^ examined the peristaltic behavior of a micropolar fluid along with a radial magnetic field. The analysis is set up for the situation when homogeneous-heterogeneous chemical reactions are taken into account. The impacts of Newtonian heating and heat source/sink are also taken into account. According to their findings, pressure gradient, pressure rise, and velocity are affected differently by the micropolar and coupling factors. The study of Asha and Deepa^11^ looked deeply into entropy generation and how thermal radiation affects the peristaltic blood transmission of a magneto-micropolar liquid in a varying channel. Low Reynolds numbers along with long wavelength approximations were used by them to conduct the analysis. According to the results, entropy generation decreases when the magnetic parameter increases, whereas it decreases as the thermal radiation parameter increases. The biomedical sciences benefit from such a result. Furthermore, it has been discovered from their results that bio-fluids like blood are better suited to the micropolar fluid model. Mahmood et al.^12^ intended to confer a brand-new boundary condition for the peristaltic transport of a micropolar fluid in an asymmetric 2D geometry. For lubrication, a power-law fluid with a thin coating film is utilized. In the presence of lubricant, they investigate the reflux criterion. According to their research, lubricant parameters have a decreasing influence on wall shear stress. The MHD movement of Williamson liquid through a non-Darcy porous medium with micro-rotation was considered by Mishra et al.^13^. To solve the ODEs, they used the shooting technique combined with the Runge–Kutta method. Their research demonstrates that the magnetic field, non-Darcy, and system characteristics all tend to lower fluid velocity.

Recently, the study of convective transport of nanofluids has gained considerable importance. In 1995, Choi^14^ conceived the idea of using nanofluids as a mode of enhancement of heat transfer of the working fluid. He gave the idea of using nanofluids as a coolant in different industrial problems. This made various authors study nanofluids and their numerous properties, a lot of useful work has been done since then^15–19^. The examination of the impact that micro-rotation creates on the peristaltic flow of a nanofluid under the influence of thermal radiation was performed by Dhanapal et al.^20^. They arrived at the conclusion that higher radiation parameter values result in increased heat flux and, hence, a lower temperature profile. Similar to this, Abou-Zeid^21^ explored the micropolar nanofluid within a conduit while taking into account the effects of viscous dissipation. The obtained results showed a higher rate of heat transfer with impacts of dissipation. The water-based peristaltic nanofluid movement with Joule heating and convective transfer of heat at the surface was examined by Hayat et al.^22^. They came to the conclusion that the velocity and temperature of the fluid decrease with the volume fraction of nanoparticles. To explore the peristaltic phenomenon, Ali et al.^23^ took into account a porous saturated channel with hydromagnetic effects. According to the Sutterby model, El-Dabe et al.^24^ study observed the peristaltic transportation of the micropolar non-Newtonian nanofluid under incompressibility conditions. They analyzed the mass and heat movement inside a two-dimensional symmetric vertical channel under the consideration of thermal radiation along with other effects and revealed that a high magnetic field has an impact on the entire analysis. Their results indicated an increase in the trapped bolus size with an increase in the three previously mentioned parameters. An electrically conducting micropolar nanofluid was studied by Mohanty et al.^25^ for its interaction with radiative heat energy and the heat source/sink. They used thermophoresis and Brownian motion to create a Buongiorno model nanofluid from their conducting fluid. The main goal of the inquiry is to analyze the irreversibility process brought on by heat transfer and entropy production. Their research's findings demonstrated that because heat processes are irreversible, the Brinkman number improves entropy. In an esophagus that has been represented mathematically as a non-uniform channel, Maraj et al.^26^ investigated the thermally driven transit of solute particles (measured in micro to nanometers) suspended in blood. Their research aims to examine the interactions between solute particles in the blood and nanoparticles in the esophagus, with the goal of giving applications for drug administration and biomedical engineering, among other things. Their investigation shows that the volumetric proportion of nanoparticles is responsible for the temperature increase and fluid flow acceleration. Sara et al.^27^ present the inaugural determination of the slip coefficient for tubular carbon structures, which were synthesized through chemical vapor deposition on a porous alumina substrate featuring nominal pore diameters of 200 nm. A consistent carbonaceous coating, measuring 20−30 nm in thickness, was uniformly developed over the pores. Karniadakis et al.^28^. Encapsulates the foundational principles and simulation methodologies related to microflows and nanoflows. Further recent literature related to the topic is given in Refs.^29–33^.

Electro-osmotic flow holds significant importance in various micro-channel processes and is particularly crucial in biotechnology applications characterized by inherent charge imbalances. This phenomenon finds notable applications in diverse areas such as tissue culture, cell scaffolding systems, pharmacodynamics, and nanoscale medical devices^34^. In a study conducted by Tripathi et al.^35^, the electro-thermal peristaltic transport of nanofluid in a finite microchannel was explored, incorporating the Chakraborty-Roy nanofluid electrokinetic formulation. Additionally, Ijaz et al.^36^ delved into the impact of electro-osmosis on bio-nanofluid containing non-spherical particles within a curved channel. Their computational findings demonstrated that the introduction of blade-shaped particles resulted in an enhanced heat transfer. In a recent investigation, Khan et al.^37^ examined the influence of radiation on electro-osmosis modulated peristaltic flow within a tapered channel, utilizing Prandtl nanofluid. Their findings revealed that isothermal lines expanded with an increase in the electro-osmotic parameter.

The current inquiry is conducted to present the concept and mathematical characteristics of Electroosmotically connected micropolar fluids as a conclusion of the aforementioned literature. The current work focuses on the selection of a micropolar model for dispersing nano-structures to analyze the natural convection. In an asymmetric channel, we investigated the peristaltic movements of an incompressible micropolar nanofluid. Low Reynolds number along with a long wavelength are the foundations upon which analysis of flow has been built. We established answers for the axial velocity, micro rotation component, and stream function after determining the precise analytical solutions to the situation at hand. Also derived are expressions for the shear stresses. Numerical integration is used to examine the influence of relevant parameters on pressure rise. The phenomenon of entrapment is examined in further detail. The outcomes for Newtonian fluid and micropolar fluid are contrasted. Moreover, the paper is presented in the following manner. The basic fluid model is described in Sects. "Base fluid model", "Flow equations" and "Thermal properties of base model", followed by the proposed micropolar nanofluid's governing equations and the thermal characteristics of the working fluids. In Sect. "Numerical solutions", we provided the precise answers to the equations in question. With regard to the heat transmission performance of a micropolar nanofluid using constant solid nanoparticle volume fractions and the selection of nanoparticles (Copper and Silver), a graphical examination of the profile of velocity and pressure parameters is discussed in Sect. "Results and discussions".

## Base fluid model

In the absence of body forces and the body couple and the governing equations for the steady flow of an incompressible micropolar fluid, are given by Refs.^4–9,19,20^

1 $$\nabla .\overline{\mathbf{v} }=0,$$

2 $${\rho }_{nf}\left(\bar{v}.\nabla \right)\bar{v}=-\nabla \bar{P}+k\nabla \times \bar{w}+\left({\mu }_{nf}+k\right){\nabla }^{2}\bar{v}+{\left(\rho \beta \right)}_{nf}g\left(\bar{T}-{\bar{T}}_{0}\right)+{\rho }_{e}E,$$

3 $${\rho }_{nf}j\left(\bar{v}.\nabla \right)\bar{w}=-2k\bar{w}+k\nabla \times \bar{v}-{\gamma }_{nf}\left(\nabla \times \nabla \times \bar{w}\right)+(\alpha +{\beta }_{1}+{\gamma }_{nf})\nabla \left(\nabla .\bar{w}\right),$$

4 $${\left(\rho {c}_{p}\right)}_{nf}\left(\bar{v}.\nabla \right)\bar{T}={K}_{nf}\hspace{0.33em}{\nabla }^{2}\bar{T}+\tau .{L}^{t}+{Q}_{0}.$$

Further, the material constants satisfy the following inequalities.

5 $$2{\mu }_{nf}+k\ge 0, \, k\ge 0, \, 3\alpha +{\beta }_{1}+{\gamma }_{nf}\ge 0, \, {\gamma }_{nf}\ge \left|{\beta }_{1}\right|.$$

## Flow equations

**Figure 1:**
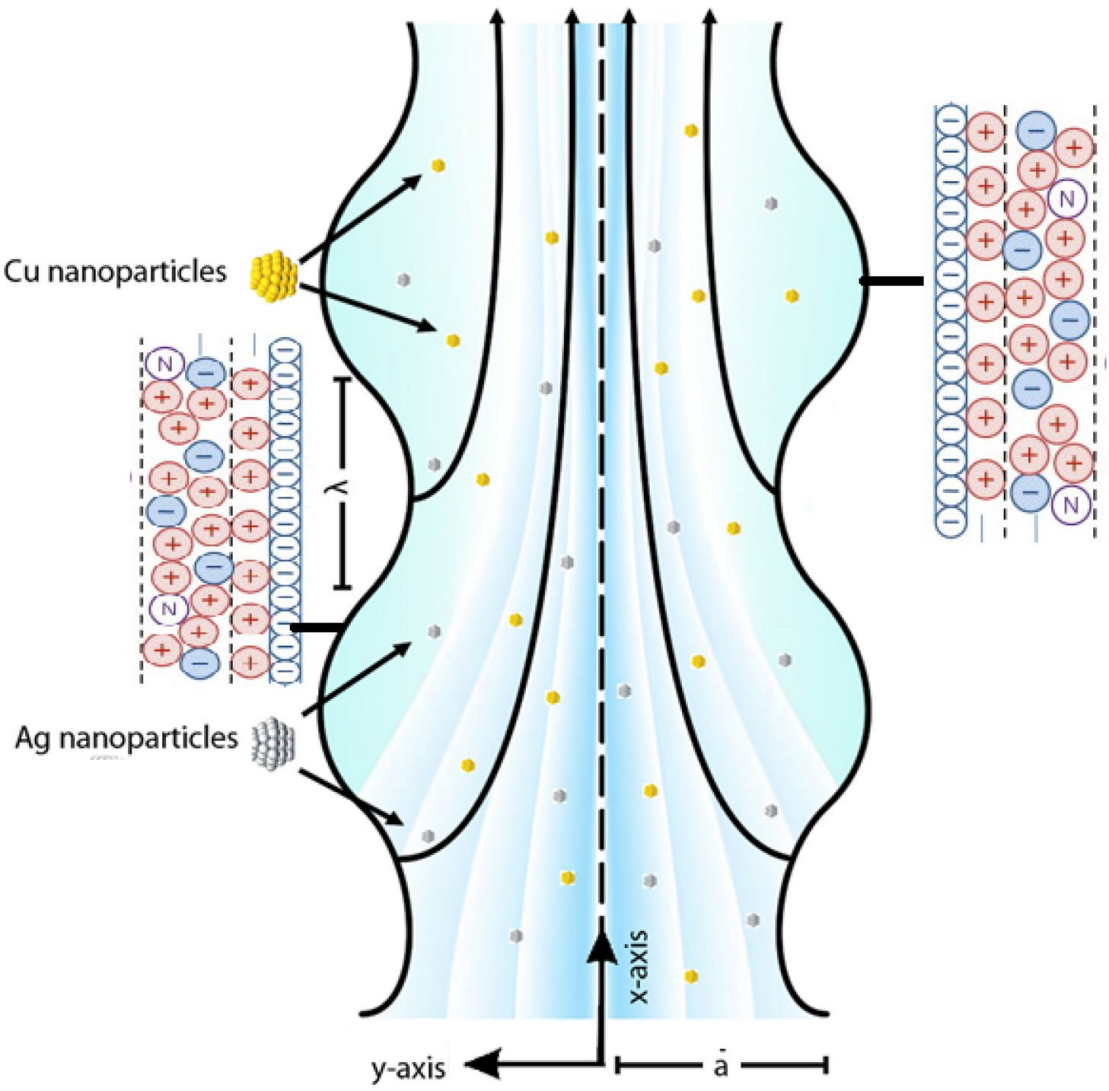
Geometry of the problem.

Let us observe the natural convective peristaltic flow under the incompressibility condition. The micropolar nanofluid is considered in a uniform channel for the analysis. The symmetrical conduit of length $L$ containing water-based micropolar nanoliquid along with natural convection is considered. As shown in Fig. 1, the coordinates are selected in a way that put $\overline{X }$ along the lower horizontal boundary and $\overline{Y }$ along the left upright wall.

Let $\overline{{T }_{0}}$ be the temperature given to the wall of the channel. The non-conductive boundary is assumed and the wall shape is defined as

6 $$\overline{h}\left( {\overline{X},\,t} \right) = a + b\sin \left( {\frac{2\pi }{\lambda }\left( {\overline{X} - ct} \right)} \right),\,$$

where $b$ & $\lambda$ show the wave amplitude and length between consecutive crests or troughs of waves, $a$ defines distance between the boundaries where propagation wave is shown by $c$. For defining time symbol $t$ is used, and $\overline{X }$ shows wave propagation direction. The transformations connecting both frames are:

7 $$\overline{x} = \overline{X} - ct,\,\;\overline{y} = \overline{Y},\,\;\overline{u} = \overline{U} - c,\,\;\overline{v} = \overline{V},\,\;\overline{P}\left( {\overline{x}} \right) = \overline{P} \left( {\overline{X},\,t} \right).$$

For the flow being taken, the velocity field is ${\overline{\mathbf{v}}}$ $=$ $(\overline{u},\,\overline{v},\,0)$ and micro rotation vector is ${\overline{\mathbf{w}}}$ $= (0,\,0,\,\overline{w})$. Then, the flow equations in component form can be written as follows Refs.^4–9,19,20^:

8 $$\frac{\partial \bar{u}}{\partial \bar{x}}+\frac{\partial \bar{v}}{\partial \bar{y}}=0$$

9 $${\rho }_{nf}\left(\bar{u}\frac{\partial \bar{u}}{\partial \bar{x}}+\bar{v}\frac{\partial \bar{u}}{\partial \bar{y}}\right)=-\frac{\partial \bar{P}}{\partial \bar{x}}+\left({\mu }_{nf}+k\right)\left(\frac{{\partial }^{2}\bar{u}}{\partial {\bar{x}}^{2}}+\frac{{\partial }^{2}\bar{u}}{\partial {\bar{y}}^{2}}\right)+k\frac{\partial \bar{w}}{\partial \bar{y}}+{\left(\rho \beta \right)}_{nf}g\left(\bar{T}-{\bar{T}}_{0}\right)+{\rho }_{e}{E}_{\bar{x}},$$

10 $$\rho_{nf} \left( {\overline{u}\frac{{\partial \overline{v}}}{{\partial \overline{x}}} + \overline{v}\frac{{\partial \overline{v}}}{{\partial \overline{y}}}} \right) = - \frac{{\partial \overline{P}}}{{\partial \overline{y}}} + \left( {\mu_{nf} + k} \right)\,\left( {\frac{{\partial^{2} \overline{v}}}{{\partial \overline{x}^{2} }} + \frac{{\partial^{2} \overline{v}}}{{\partial \overline{y}^{2} }}} \right) - k\frac{{\partial \overline{w}}}{{\partial \overline{x}}},\,$$

11 $$j\rho_{nf} \left( {\overline{u}\frac{{\partial \overline{w}}}{{\partial \overline{x}}} + \overline{v}\frac{{\partial \overline{w}}}{{\partial \overline{y}}}} \right) = - 2k\overline{w} + \gamma_{nf} \left( {\frac{{\partial^{2} \overline{w}}}{{\partial \overline{x}^{2} }} + \frac{{\partial^{2} \overline{w}}}{{\partial \overline{y}^{2} }}} \right) + k\left( {\frac{{\partial \overline{v}}}{{\partial \overline{x}}} - \frac{{\partial \overline{u}}}{{\partial \overline{y}}}} \right),\,$$

12 $${\left(\rho {c}_{p}\right)}_{nf}\left(\bar{u}\frac{\partial \bar{T}}{\partial \bar{x}}+\bar{v}\frac{\partial \bar{T}}{\partial \bar{y}}\right)={K}_{nf}\left(\frac{{\partial }^{2}\bar{T}}{\partial {\bar{y}}^{2}}+\frac{{\partial }^{2}\bar{T}}{\partial {\bar{x}}^{2}}\right)+\left({\mu }_{nf}+k\right)\left({\left(\frac{\partial \bar{u}}{\partial \bar{x}}\right)}^{2}+\bar{2}{\left(\frac{\partial \bar{v}}{\partial \bar{y}}\right)}^{2}+{\left(\frac{\partial \bar{u}}{\partial \bar{x}}+\frac{\partial \bar{v}}{\partial \bar{y}}\right)}^{2}\right)+{Q}_{0}.$$

where $\overline{u }$ and $\overline{v }$ are the velocity components along $\overline{x }$ and $\overline{y }$ axes, $\overline{T }$ shows fluid temperature whereas, g stands for the acceleration due to gravity, ${\rho }_{nf}$ is the effective, density, ${\mu }_{nf}$ is the effective dynamic viscosity,${\gamma }_{nf}$ is the spin-gradient viscosity, $j$ is the micro-inertia density and $k$ is a constant. For further analysis, we use the following non-dimensional variables and parameters:

13 $$\begin{aligned} y = & \frac{{\overline{y}}}{a},\, \, x = \frac{{\overline{x}}}{\lambda },\, \, t = \frac{{c\overline{t}}}{\lambda },\, \, v = \frac{{\lambda \overline{v}}}{ac},\, \, \varphi = \frac{b}{a},\, \, u = \frac{{\overline{u}}}{c},\, \, w = \frac{a}{c}\overline{w},\, \\ P = & \frac{{\overline{P}a^{2} }}{{\lambda c\mu_{{_{f} }} }},\, \, h = \frac{{\overline{h}}}{a},\, \, Re = \frac{\rho ca}{{\mu_{{_{f} }} }},\, \, \delta = \frac{a}{\lambda },\, \, j = \frac{{\overline{j}}}{{a^{2} }},\, \, \theta = \frac{{\overline{T} - \overline{T}_{0} }}{{\overline{T}_{0} }},\, \\ \beta = & \frac{{Q_{0} a^{2} }}{{k_{f} \overline{T}_{0} }},\, \, G_{r} = \frac{{g\alpha a^{2} \overline{T}_{0} \rho_{f} }}{{c\mu_{f} }}. \\ \end{aligned}$$

With the non-dimensional variables, Eqs. (7), (8), (9), (10) and (11) take the following form:

14 $$\textit{Re}\delta \left(u\frac{\partial }{\partial x}+v\frac{\partial }{\partial y}\right)u=-\frac{\partial P}{\partial x}+\left(\frac{{\mu }_{nf}}{{\mu }_{f}}+\frac{1}{N}\right)\left({\delta }^{2}\frac{{\partial }^{2}u}{\partial {x}^{2}}+\frac{{\partial }^{2}u}{\partial {y}^{2}}\right)+\frac{1}{N}\frac{\partial w}{\partial y}+{G}_{r}\frac{{\left(\rho \beta \right)}_{nf}}{{\left(\rho \beta \right)}_{f}}\theta +{U}_{HS}\frac{{\partial }^{2}E}{\partial {y}^{2}},$$

15 $$Re\delta^{3} \left( {u\frac{\partial }{\partial x} + v\frac{\partial }{\partial y}} \right)\,v = - \frac{\partial P}{{\partial y}} + \frac{{\delta^{2} }}{{\left( {1 - N} \right)}}\left( { - N\frac{\partial w}{{\partial x}} + \delta^{2} \frac{{\partial^{2} v}}{{\partial x^{2} }} + \frac{{\partial^{2} v}}{{\partial y^{2} }}} \right),\,$$

16 $$Re\delta j\left( {u\frac{\partial }{\partial x} + v\frac{\partial }{\partial y}} \right)\,w = - 2w + \left( {\delta^{2} \frac{\partial v}{{\partial x}} - \frac{\partial u}{{\partial y}}} \right) + \left( {\delta^{2} \frac{{\partial^{2} w}}{{\partial x^{2} }} + \frac{{\gamma_{{_{nf} }} }}{{\mu_{{_{f} }} }}\Omega \frac{{\partial^{2} w}}{{\partial y^{2} }}} \right)\,,$$

17 $$\textit{Re}\delta Pr\left(u\frac{\partial }{\partial x}+v\frac{\partial }{\partial y}\right)\theta =\frac{{\partial }^{2}\theta }{\partial {y}^{2}}+\frac{{K}_{f}}{{K}_{nf}}\left(\frac{{\mu }_{nf}}{{\mu }_{f}}+\frac{1}{N}\right){\left(\frac{\partial u}{\partial y}\right)}^{2}+\frac{{K}_{f}}{{K}_{nf}}\beta ,$$

where $N = \frac{{\mu_{{_{f} }} }}{k}$ is the coupling number and $\Omega = \frac{{\mu_{{_{f} }} }}{{ka^{2} }}$ is a micropolar parameter, $\alpha$ and $\beta_{1}$, do not appear in the governing equation as the micro rotation vector is solenoidal. Under the assumptions of long wavelength, neglecting inertia terms $\left( {Re = 0} \right),$ we have the following system of equations

18 $$0=-\frac{\partial P}{\partial x}+\left(\frac{{\mu }_{nf}}{{\mu }_{f}}+\frac{1}{N}\right)\left(\frac{{\partial }^{2}u}{\partial {y}^{2}}\right)+\frac{1}{N}\frac{\partial w}{\partial y}+{G}_{r}\frac{{\left(\rho \beta \right)}_{nf}}{{\left(\rho \beta \right)}_{f}}\theta +{U}_{HS}\frac{{\partial }^{2}E}{\partial {y}^{2}},$$

19 $$0 = - \frac{\partial P}{{\partial y}},\,$$

20 $$0 = - 2w + \left( { - \frac{\partial u}{{\partial y}}} \right) + \left( {\frac{{\Omega \gamma_{{_{nf} }} }}{{\mu_{{_{f} }} }}\frac{{\partial^{2} w}}{{\partial y^{2} }}} \right)\,$$

21 $$0=\frac{{\partial }^{2}\theta }{\partial {y}^{2}}+\frac{{K}_{f}}{{K}_{nf}}\left(\frac{{\mu }_{nf}}{{\mu }_{f}}+\frac{1}{N}\right){\left(\frac{\partial u}{\partial y}\right)}^{2}+\frac{{K}_{f}}{{K}_{nf}}\beta ,$$

22 $$\frac{{\partial }^{2}E}{\partial {y}^{2}}={\upkappa }^{2}\left(\frac{{{\text{n}}}^{-}-{{\text{n}}}^{+}}{2}\right),$$

where ${U}_{HS}$ designates the Helmholtz-Smoluchowski velocity or electroosmotic velocity parameter, $Pr$ the Prandtl number, $\theta$ the dimensionless temperature parameter, and $\kappa$ is the ratio of the characteristic traverse length to the Debye length parameter. The local ionic distribution of ionic species can be specified by linearized Boltzmann distribution for low zeta potential which accurately estimates the electric potential established in the fluid medium without increasing the complexity of the flow problem as for most of the electrolyte solution, the generated electric potential lies in the range less than or equal to 25mV.

23 $${n}^{\pm }={e}^{\mp E},$$

After the linearized Poisson-Boltzmann paradigm^31^ as:

24 $$\frac{{\partial }^{2}E}{\partial {y}^{2}}={\kappa }^{2}{\text{sinh}}\left(E\right),$$

which is further simplified under Debye-Hückel approximation^31^ i.e. ${\text{sinh}}\left(\varphi \right)\approx \varphi$ as:

25 $$\frac{{\partial }^{2}E}{\partial {y}^{2}}={\kappa }^{2}E.$$

wing boundary conditions:

26 $$\frac{\partial u}{\partial y}=0, \frac{\partial w}{\partial y}=0, \frac{\partial \theta }{\partial y}=0, \frac{\partial E}{\partial y}=0, \text{ at }y=0,$$

27 $$u=-1, w=0, \theta =0, \, E=\xi , \text{ at }y=h\left(x\right)=1+\varepsilon \,{\sin}2\pi x.$$

## Thermal properties of base model

$\gamma_{{_{nf} }}$ has the form shown in^19,20^, which is as follows:

28 $${\gamma }_{nf}=\left({\mu }_{nf}+\frac{k}{2}\right)j.$$

The nanofluid's effective density is given as follows:

29 $${\rho }_{nf}=\left(1-\varphi \right){\rho }_{f}+\varphi {\rho }_{p}.$$

The nanofluid's thermal expansion coefficient can be calculated as follows:

30 $${\left(\rho \beta \right)}_{nf}=\left(1-\varphi \right){\left(\rho \beta \right)}_{f}+\varphi {\left(\rho \beta \right)}_{p}.$$

The experimentally determined equations for the effective viscosity $\mu_{nf}$ and thermal conductivity $k_{nf}$ of the nanofluid are as follows^30^:

31 $${\mu }_{nf}=\frac{{\mu }_{f}}{{\left(1-\varphi \right)}^{2.5}};\hspace{0.33em}{k}_{nf}={k}_{f}\left(\frac{{k}_{p}+2{k}_{f}+2\varphi \left({k}_{p}-{k}_{f}\right)}{{k}_{p}+2{k}_{f}-2\varphi \left({k}_{p}-{k}_{f}\right)}\right),\text{ Cu Nanoparticles }:$$

$${\mu }_{nf}={\mu }_{f}\left(1.005+0.497\varphi -0.1149{\varphi }^{2}\right)$$

32 $${k}_{nf}={k}_{f}\left(0.9508+0.9692\varphi \right),\text{ Ag Nanoparticles.}$$

## Numerical solutions

**Figure 2:**
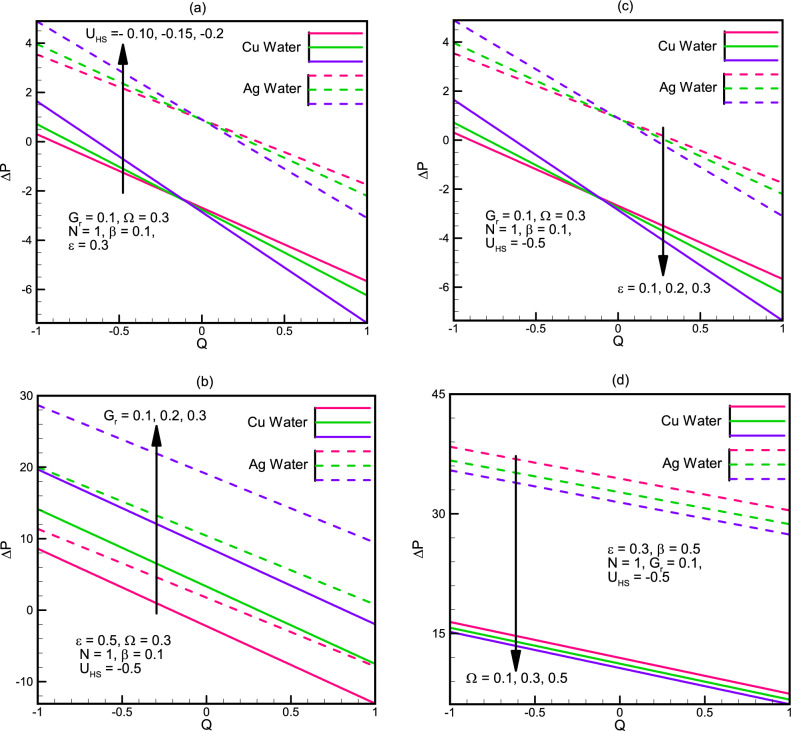
(**a**) Pressure rise Δ*P* versus flow rate for different values of *U*_*HS*_. (**b**) Pressure rise Δ*P* versus flow rate for different values of *G*_*r*_. (**c**) Pressure rise Δ*P* versus flow rate for different values of ε. (**d**) Pressure rise Δ*P* versus flow rate for different values of Ω.


To solve the simplified system defined in Eqs. (18), (19), (20), (21), (22), (23), (24) and (25) owing to boundary conditions in Eqs. (26) and (27). Computational software MATLAB is utilized, and numerical results are obtained by employing inbuilt solver based of three stage Lobatto IIIa formula known as bvp4c. Figure 2 illustrate the complete flow chart of bvp4c algorithm.

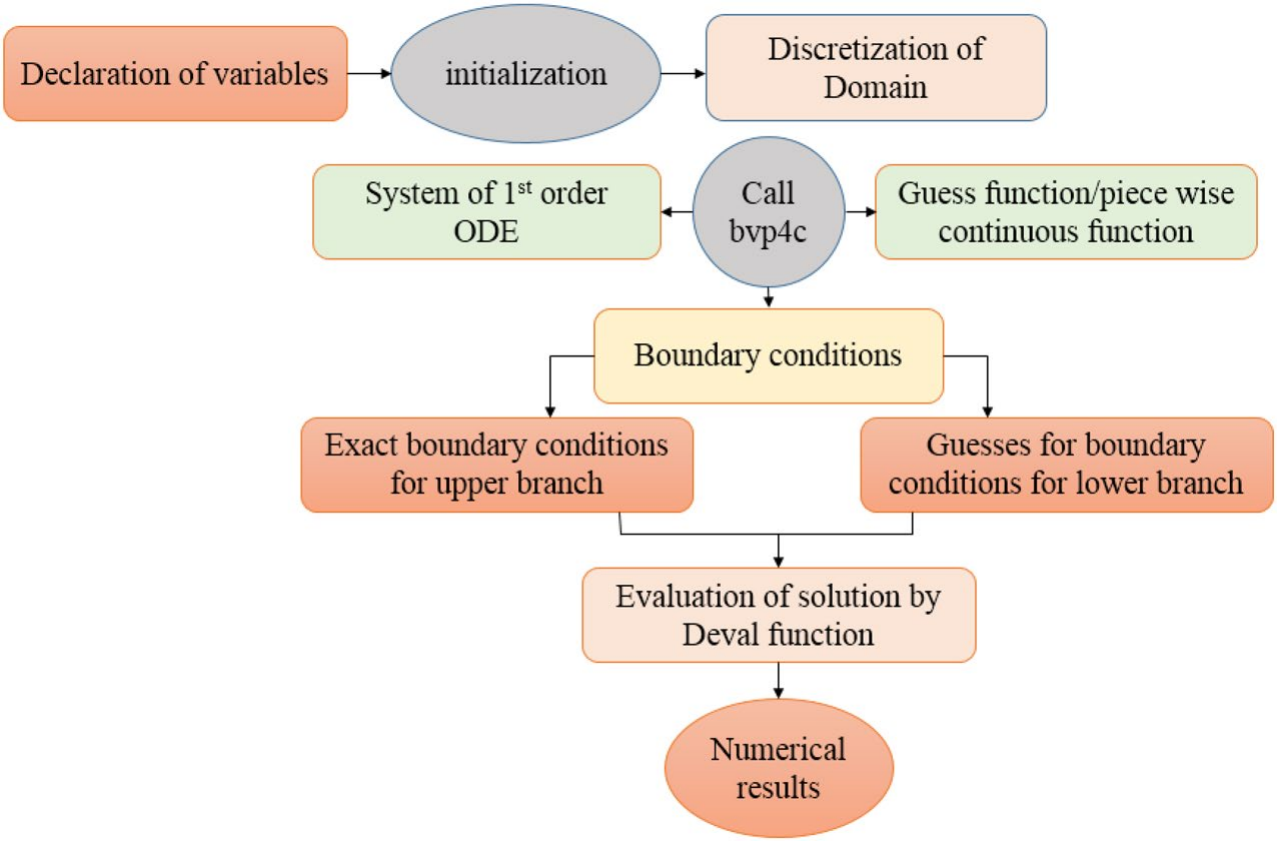


The pressure rise expression is given as follows:

33 $$\Delta P = \int\limits_{0}^{1} {\frac{{dp}}{{dx}}dx} .$$

Skin friction coefficient is given as follows

34 $${\left.{C}_{f}=-\left(\frac{{\mu }_{nf}}{{\mu }_{f}}+\frac{1}{N}\right)\left(\frac{{\partial }^{2}u}{\partial {y}^{2}}\right)\right|}_{y=0}$$

Heat transfer rate is given as follows

35 $${\left.{N}_{u}=-\frac{{K}_{nf}}{{K}_{f}}\frac{\partial \theta }{\partial y}\right|}_{y=0}$$

## Results and discussions

The precise solutions found in the former section are displayed graphically to analyze the effects of the physical parameters on the magnitude of the temperature, pressure and velocity profiles. Figure 2a–d represent the pressure rise affected by different physical constraints. From the graphs, we perceive that the greater the ${U}_{HS}$ the Helmholtz-Smoluchowski velocity the greater the pressure rise but the peak amplitude $\varepsilon ,$ which also increases with the wave amplitude $\beta ,$ increases the pressure rise in the peristaltic pumping region and decreases the pressure rise in the augmented pumping region. Also with the increase in the Grashoff number $Gr$ the pressure rise also increases, this indicates that higher the buoyancy forces in comparison to the viscous forces, more pressure rise is observed and vice versa. Also we observe that the micropolar parameter is inversely proportional to the pressure rise and that the pressure rise for $Ag$-water is greater than $Cu$-water in all cases.

**Figure 3:**
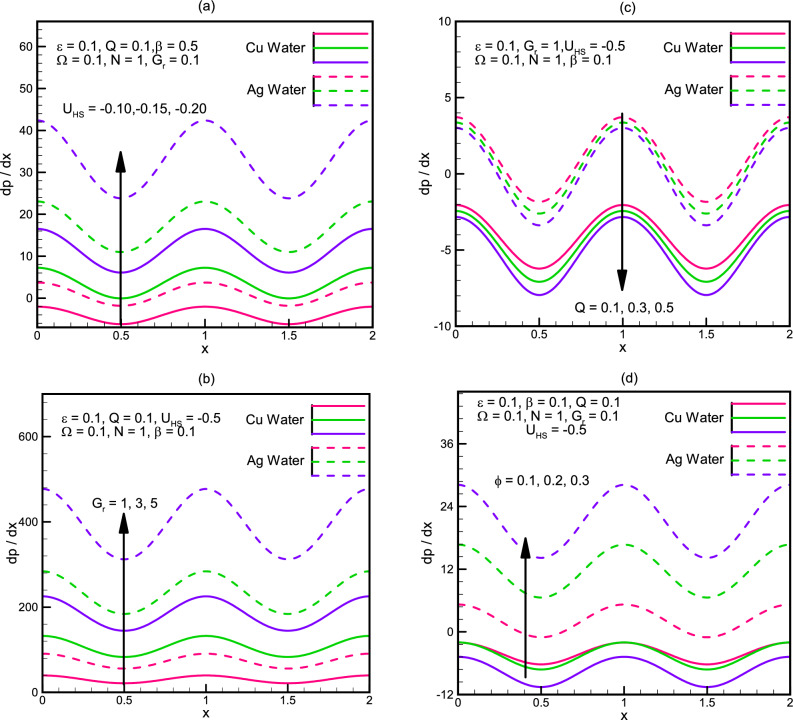
(**a**) Pressure gradient $\frac{dp}{dx}$ versus flow rate for different values of ${U}_{HS}$. (**b**) Pressure gradient $\frac{dp}{dx}$ versus flow rate for different values of ${G}_{r}$. (**c**) Pressure gradient $\frac{dp}{dx}$ versus flow rate for different values of $Q$. (**d**) Pressure gradient $\frac{dp}{dx}$ versus flow rate for different values of $\varnothing$.

Pressure gradient is graphically analyzed in Fig. 3a–d$.$ We observe that the pressure gradient, for Cu/Ag nanofluid is directly proportional to the ${U}_{HS}$ the Helmholtz-Smoluchowski velocity as well as the Grashoff number $Gr$. It is inversely proportional to the mean flow rate $Q$. However, the behavior of pressure gradient for the variation of the solid nanoparticle volume $\varphi$ fraction is not same for Cu-water and Ag-water, Fig. 3d depicts that the pressure gradient decreases with an increase in $\varphi$ for Cu-water fluid, and pressure gradient increases with an increase in ϕ for Ag-water fluid. Similar to pressure rise, the pressure gradient for Ag-water in all cases is greater than the pressure gradient.

**Figure 4:**
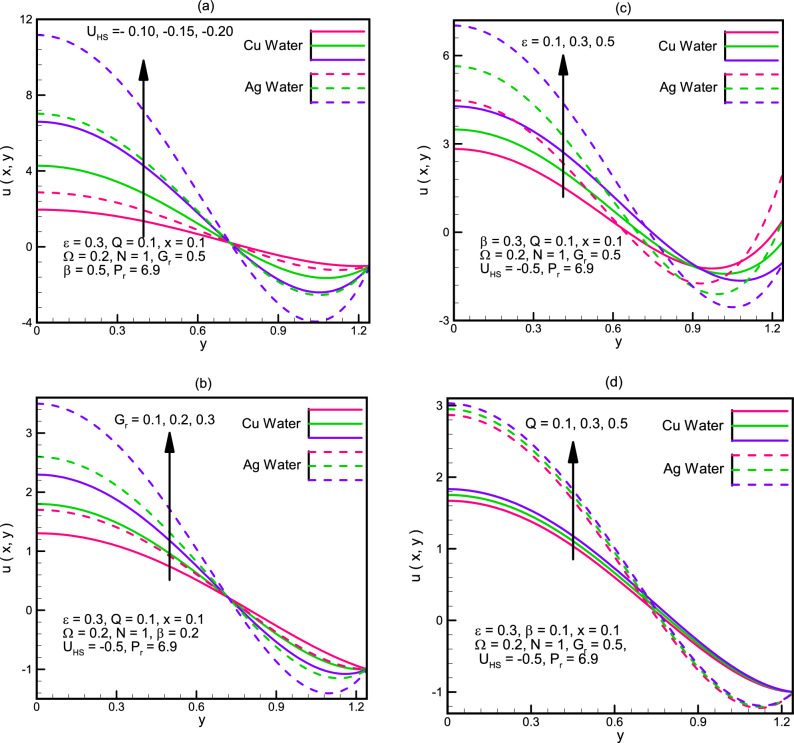
(**a**) Velocity u(x,y) for different values of U_HS. (**b**) Velocity u(x,y) for different values of G_r. (**c**) Velocity u(x,y) for different values of ε. (**d**) Velocity u(x,y) for different values of Q.

**Figure 5:**
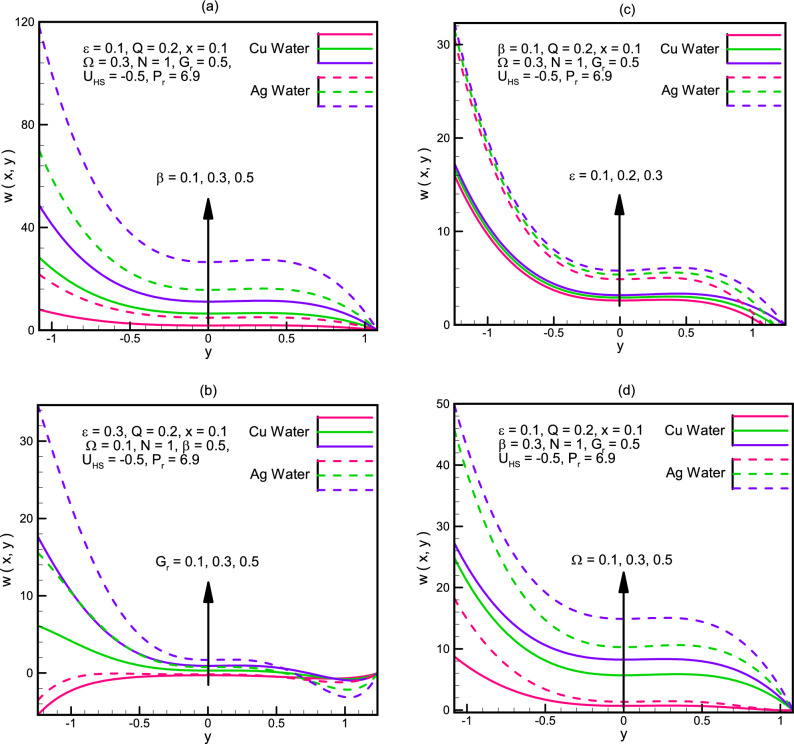
(**a**) Velocity W(x,y) for different values of β. (**b**) Velocity W(x,y) for different values of G_r. (**c**) Velocity W(x,y) for different values of ε. (**d**) Velocity W(x,y) for different values of Ω.

Velocity along the channel $u\left(x,y\right)$ is shown in Fig. 4a–d$.$ Graphical demonstration depicts that the velocity along the channel increases for $0<x<0.8$ and it decreases for $0.8<x<1.25$ for increasing values of ${U}_{HS},{G}_{r},\varepsilon ,Q$. We see that the change in the velocity for Ag-water is more rapid as compared to the change in Cu-water. Figure 5a–d illustrate the graphs of velocity $w\left(x,y\right)$ in the y-direction. We notice that the behavior of velocity is not uniform, for small wave amplitude $\beta ,$ the values of Cu-water and Ag-water coincide but as the wavelength starts increasing, the difference between Cu-water and Ag-water fluid starts increasing with Ag-water gaining more rapid growth. Similar behavior is seen for Gr, ϵ and Ω.

**Figure 6:**
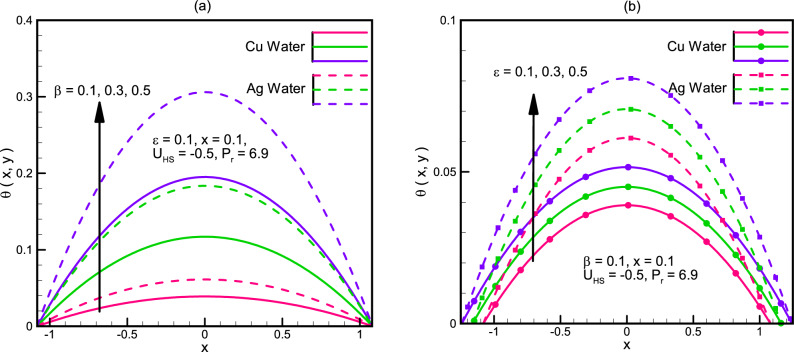
(**a**) Temperature profile θ(x,y) for different values of β. (**b**) Temperature profile θ(x,y) for different values of ε.

The graphs of the temperature distribution are displayed in Fig. 6a,b. We notice that temperature in case of Ag-water is more as compared to temperature in case of Cu-water. Also the rate of change of temperature with respect to the increase in $\beta ,\varepsilon$ is faster for Ag-water than that of Cu-water.

**Figure 7:**
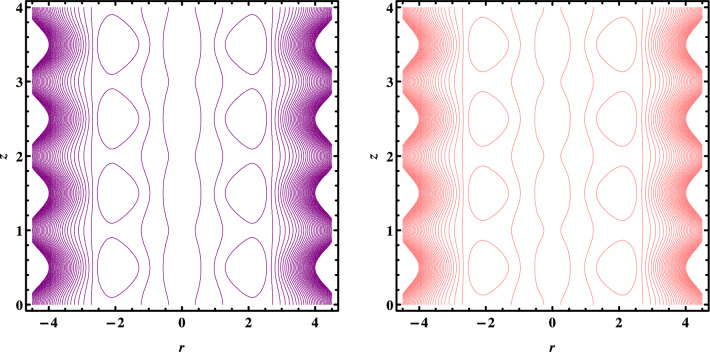
Streamlines for $w\left(r,z\right)$ when ${G}_{r}=\mathrm{1,2}.$

**Figure 8:**
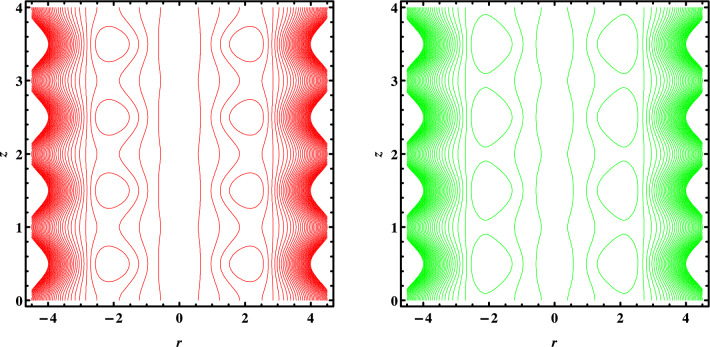
Streamlines for $w\left(r,z\right)$ when $\beta =\mathrm{9,10}.$

**Figure 9:**
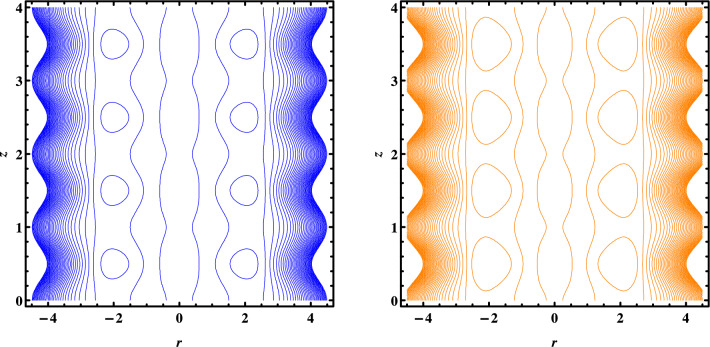
Streamlines for $w\left(r,z\right)$ when ${U}_{HS}=-0.10,-0.15.$

Figures 7, 8 and 9 depict the trapping phenomena for various flow parameters; it can be seen that as the Grashoff number $Gr$ rises, the size of the trapped bolus begins to diminish. The trapped bolus begins to grow in size when ${U}_{HS}$ the Helmholtz-Smoluchowski velocity and heat absorption parameter increase which is the opposite of the tendency seen for those two variables. Table 1. Present thermophysical properties of water and different kind of nanofluids. Table 2. Give comparison of present results with existing literature. Tables 3 and 4 gives the numerical values of Skin friction coefficient and heat transfer rate for different flow parameters. It is seen that the skin friction coefficient increases with the increase in flow rate Q and nanoparticles volume fraction $\varphi$ and decreases with the increase in Coupling number N, and heat transfer rate increases with increase in β and decreases with the increase in $\varphi .$

**Table 1:** Thermophysical properties of water and different kind of nanofluids Ref.^32^.

Type of fluid	$\rho_{p} \left( {kg/m^{3} } \right)$	$C_{p} \left( {J/kg\;K} \right)$	$k_{p} \left( {W/m\;K} \right)$	$\beta * 10^{ - 5} \left( {K^{ - 1} } \right)$
Pure water $\left( {H_{2} O} \right)$	997.1	4179	0.613	21
Alumina $\left( {Al_{2} O_{3} } \right)$	3970	765	40	0.85
Cu water (*Cu*)	8933	385	401	1.67
Titanium oxide ${\left( {TiO_{2} } \right)}$	4250	686.2	8.9538	0.9
Silver (*Ag*)	10,500	235	429	1.89

**Table 2:** Give comparison of present results with existing literature.

y	u(x,y),${G}_{r}=0,\varphi =0,{U}_{HS}=0$	Ref.^8^	Ref.^13^ when We = 0, M = 0, K = 0
0	0.79954	0.79952	0.79953
0.2	0.36363	$0.36361$	0.36365
0.4	0.75414	0.75413	0.75416
0.6	0.61863	0.61862	0.61864
0.8	0.39237	0.39236	0.39234
1.0	0.07250	0.07251	0.07251
1.2	0.01257	0.01255	0.01254

**Table 3:** Gives the numerical values of skin friction coefficient for different flow parameters.

Q	${C}_{f}$	N	${C}_{f}$	$\varphi$	${C}_{f}$
0.0	3.75204	0.2	4.21955	0.00	10.6570
0.3	4.03255	0.4	4.20631	0.05	13.3006
0.6	4.31306	0.6	4.12461	0.10	14.9787
0.9	4.59357	0.8	4.11032	0.15	15.9398
1.2	4.87408	1.0	4.01213	0.20	16.3710
1.5	5.15459	1.2	4.00134	0.25	16.4222
1.8	5.43510	1.4	3.99312	0.30	16.2211

**Table 4:** Gives the numerical values of heat transfer rate for different flow parameters.

$\beta$	${N}_{u}$	$\varphi$	${N}_{u}$
0.0	0.00000	0.0	0.06679
0.3	0.20038	0.05	0.05653
0.6	0.40076	0.10	0.04321
0.9	0.60114	0.15	0.32124
1.2	0.80153	0.20	0.22134
1.5	1.00191	0.25	0.12341
1.8	1.20228	0.30	0.02312

## Concluding remarks

Peristaltic flow of Micropolar nanofluid in a symmetric channel has been investigated with electroosmotic forces. Cu and Ag nanoparticles are utilized within water-based fluid. The core outcomes of this study can be summarized as:

- Micro rotation phenomena enhance the thermal conductivity of the fluid flow.
- It is observed that increasing ${U}_{HS}$ the Helmholtz-Smoluchowski velocity, velocity increases for both the nanoparticles.
- Pressure gradient is directly proportional to wave amplitude for both kinds of nano fluids.
- We observe that the pressure gradient, for Cu/Ag nanofluid is directly proportional to the ${U}_{HS}$ the Helmholtz-Smoluchowski velocity.
- The size of the trapped bolus rises with flow rate and heat absorption parameter.
- Non uniform velocity is observed for smaller wave amplitude.
- It notice that temperature in case of Ag-water is more as compared to temperature in case of Cu-water due to high thermal conductivity of Ag-water.
- The trapped bolus begins to grow in size when ${U}_{HS}$ the Helmholtz-Smoluchowski velocity increases.

## Data Availability

The datasets used and/or analyzed during the current study available from the corresponding author on reasonable request.

## Abbreviations

$\left(X,Y\right)$: Cartesian system of coordinates
$$\overline{T }$$: Temperature
$c$: Speed of wave propagation
$${\mu }_{nf}$$: Effective viscosity
$$Re$$: Reynold number
$j$: Micro-inertia density
$H$: Height of the channel
${K}_{nf}$: Effective thermal conductivity
w: Micro rotation vector
$\beta$: Heat absorption parameter
${\beta }_{1}$: Micro rotation vector
${\rho }_{e}$: Local electric charge density
$Q$: Dimensionless flow rate
$$\delta$$: Wave length
$\varphi$: Nanoparticle volume fraction
$$\left(u,v\right)$$: Velocity components
*g*: Gravitational forces
${\gamma }_{nf}$: Spin-gradient viscosity
$$t$$: Time
$$\lambda$$: Wavelength
$$P$$: Pressure
$$N$$: Coupling number
$$\Omega$$: Micropolar parameter
$\alpha$: Micro rotation vector
$$a$$: Channel radius
$$\rho$$: Density
${{\text{E}}}_{\bar{{\text{x}}}}$: The axial electric field
$$\Delta P$$: Pressure rise
Pr: Prandtl number
